# The Pauper and His Food

**Published:** 1895-06-01

**Authors:** 


### Modern Sociology.

### THE PAUPER AND HIS FOOD.

It was a historic moment in the history of the workhouse when Oliver Twist asked for more gruel. Oliver has been asking for more ever since, until conscientious Guardians hardly know the extent and the limits of his rights. There is no doubt that formerly the discipline of the workhouse was harsh and the food scanty; but it must be remembered that originally it was regarded as the temporary home of the able-bodied labourer, and it was necessary not to make it too attractive lest he should desire to make his residence permanent. The great abuses which had prevailed under the old poor laws made reformers perhaps too stringent in the new regulations; but it can hardly be denied by those who have any knowledge of human nature that the only way to make the average man work is to make idleness as unattractive as possible.

But the occupants of the workhouse now a days are for the most part either too old or too young to earn their living, and to these it may be desirable to display greater leniency. Allowing for all past improvidence and folly, we feel that the helplessness of old age deserves consideration. Admitting as an inevitable law that the sins of the father shall be visited on the children, it is hardly the place of human justice to inflict the visitation. It is not desirable, certainly, to make the workhouse a pleasant retreat for the unworthy; but it is possible and desirable to show in the treatment of the inmates a discrimination based on their conduct in the past. The food question has long been a vexatious one. It is probable that there are very few workhouses now where the gruel is deficient either in quality or quantity, but gruel every day is monotonous even to a palate not accustomed to dainties. Is it not possible to vary the diet of our paupers without—this is an essential point—adding to the expense of maintaining them?

Once a lady Guardian, walking through the sick ward of a workhouse, saw an old man looking with marked distaste at the beef-tea provided for his dinner. He was toothless, and could consume only liquid food. The lady asked him why he did not take the beef-tea, which, it may be noted, was perfectly satisfactory as to quality and savouriness. "Ma'am," he answered, "I've had beef-tea every day for six months, and I'm sick of it." Would it not have been as easy, and probably more economical, to have given the old man some other soup occasionally instead of beef-tea *ad nauseam?* Probably every workhouse visitor could quote parallel instances where a little thought on the part of those in authority would increase the comfort of the inmates without taking more from the ratepayers' pockets. On this point some suggestions made by Dr. Arthur Downes are well worthy of consideration. While deprecating the custom of bribing paupers to work by the bestowal of little luxuries as a reward, he admits the necessity of varying the food according to the employment. The pauper should not have the option to work or remain idle according as he may choose; that is a point for the master of the workhouse to decide; but if he is set to work, that fact must be borne in mind in settling his diet. Thus for old people the chief point to be regarded is that the food be easy of digestion. A large quantity is not necessary, but consideration must be had to the waning activity of the bodily organs. For the able-bodied a more abundant, but coarser, dietary is suitable. If the food is nourishing it need not be of the daintiest kind. Those who are to consume it are, presumably, strong and healthy. For children, in the workhouse as elsewhere, nourishing, muscle-forming food must be supplied, with meals more frequent than those of adults. It may be said that the children of the same class outside the workhouse get no such sufficiency. That may be, but the fact is no argument against giving the children under the Guardians' care food enough, and of the right sort. It may be permissible to give to the adult and able-bodied, who have probably been driven to the workhouse through their own follies or vices, to just such a scale of living as the poorest unskilled labour can obtain outside; but children should be given that chance to fight the battle of life fairly and escape pauperism which only good health can give.

Dr. Downes very wisely advises that the food in workhouses should be varied according to the season of the year. The food which the system craves for in winter is unsuitable for summer. "In summer," says Dr. Downes, "the bread might, perhaps, be somewhat reduced, and fresh vegetables and fruit should be given. Dry peas and other dry legumes in soup, &c., are better suited to winter than to summer; the same remark applies to oatmeal, and the children's porridge might with advantage during the hot months be replaced by bread and butter and milk, or tea made with equal parts milk. On the other hand, in winter the allowance of bread and fatty foods should properly be increased." This is common-sense on the food question, and the advice might properly be carried out elsewhere than in the workhouse. To facilitate cooks in preparing diets, Dr. Downes gives recipes for the various dishes that form the workhouse bill of fare, with the nutritive value of the nitrogenous and carbonaceous elements in each. But he does not make the blunder common to the scientific and non-practical cook, for he says, "It must always be remembered that nutritive value is not the only consideration; fitness and digestibility of food, as well as variety, both in kind and in cooking, must equally be taken into account for each class of inmates. Palatable service of meals is also important. Due care should be taken that cooked food shall be served hot, and that meat shall be carved in fairly cut slices."

Some people will say that this is pampering the pauper. Not necessarily. People who have lived in one room all their lives have at least had their food hot. And it must be remembered that pauperism, though certainly no merit, is not a crime. Accidents which could not be foreseen occasionally force the most worthy into the workhouse, and there are many whose labour, however steady and continuous, does not enable them to lay by for old age. To treat these like the vicious and shiftless, even to force the two into unwilling companionship is a wrong. Conscious of this the Guardians of the Sheffield Union have divided their paupers into four classes, of which the first are treated as if they were inmates of some cottage almshouse. Old couples are to have a room to themselves, and are encouraged to make it homelike with plants and little ornaments and bits of furniture; they are even to be allowed pet animals, and are encouraged to cultivate garden plots for their own use. Even some little cooking may be done in their own room, and everything savouring of the workhouse is to be kept in the background. But this is only for the respectable. A second grade has fewer privileges; for a third, ordinary workhouse treatment is considered good enough, while for the fourth, whose pauperism is directly due to vice, there is still harder measure, and nothing is given that would not be earned by the poorest labour in the world outside.

